# Unraveling Population Trend of a Critically Endangered Freshwater Crocodylian, Gharial ( *Gavialis gangeticus* ) in the National Chambal Sanctuary, India

**DOI:** 10.1002/ece3.72643

**Published:** 2025-12-10

**Authors:** Surya Prasad Sharma, Suyash Katdare, Ruchi Badola, Syed Ainul Hussain

**Affiliations:** ^1^ Wildlife Institute of India Dehra Dun Uttarakhand India

**Keywords:** Chambal, grow‐and‐release, habitat protection, population recovery, protected area

## Abstract

The gharial ( *Gavialis gangeticus* ), an endemic freshwater crocodylian species, has shown signs of recovery following a severe population decline, primarily due to concerted conservation efforts initiated in the mid‐1970s. However, despite decades of sustained conservation initiatives, critical information on population trends, nesting success, and winter habitat use remains lacking, which is essential for guiding and refining future conservation strategies. The present study assesses the population trend, size class composition, nesting success and factors influencing gharial distribution in the National Chambal Sanctuary, which harbours ≈80% of the global gharial population. The population trend analysis indicated a positive growth rate ($\bar{r}$ = 0.032) and a finite rate of population increase (*λ* = 1.032), reflecting a steady increase in the gharial population in the Sanctuary. Nesting efforts also showed an increasing trend, from 402 nests in 2017 to 486 nests in 2019. Habitat use analysis revealed a preference for sandy substrate and a negative association with clay and rocky substrates, suggesting habitat selectivity that influences gharial distribution across the river. Overall, findings highlight the urgent need to reassess and strengthen current gharial conservation programs by integrating habitat‐specific management, enhancing protection of prime nesting and basking sites, and sustainable use of river resources.

## 1 Introduction

The gharial ( *Gavialis gangeticus* Gmelin, 1789) is a freshwater crocodylian endemic to the Indian subcontinent (Hussain 1999). Historically, gharial was distributed across major river systems, including the Indus, Ganga, Mahanadi, Brahmaputra, Kaladan, and Irrawaddy Rivers, spanning present‐day Pakistan, India, Nepal, Bangladesh, Bhutan, and Myanmar (Hussain 1991). However, by the mid‐20th century, gharial experienced severe population and range decline in the early 20th century due to hunting, poaching, medicinal use, fishing net mortality, and habitat loss and destruction from riparian agriculture, sand mining, and altered water regimes (Choudhury and Chowdhury 1986; Hussain 2009; Katdare et al. 2011; Panda et al. 2023; Whitaker and Daniel 1980). Concern over the alarming decline prompted conservationists to initiate immediate interventions, leading to the inclusion of all three crocodylians in Schedule I of the Indian Wild Life (Protection) Act, 1972, prohibiting poaching, hunting, and trade (Singh 1978). Subsequently, the Government of India, with support from the Food and Agriculture Organization (FAO) of the United Nations Development Program (UNDP), launched a dedicated conservation and management initiative, “Project Crocodile,” in 1975 (Bustard 1976). The primary goal of the initiative was to facilitate the rapid recovery of these species through grow‐and‐release programs (Choudhury and Chowdhury 1986; Singh 1976). Under the grow‐and‐release program, eggs collected from the wild were hatched and reared in rehabilitation centers. Once the individuals reached a length of approximately 1.2 m, they were translocated into suitable habitats within the newly established protected areas (Bustard 1976; Sharma et al. 2021). Since the initiation of the program, more than 5000 gharials have been released over the past four decades into more than 12 rivers across India, with over 3500 individuals released into the Chambal and Girwa rivers (Whitaker 2007).

Although the program was aimed at restoring all three crocodylian species, the gharial, owing to its severely depleted population and unique evolutionary history, was given conservation priority, and suitable habitats were designated as Wildlife Sanctuaries to support its recovery (Choudhury and Chowdhury 1986; Rao and Choudhury 1992). The National Chambal Sanctuary (NCS) in the Chambal River, along with Satkosia Gorge Wildlife Sanctuary in the Mahanadi and Katerniaghat Wildlife Sanctuary in Girwa, has been central to the gharial recovery since the inception of the program (Sharma et al. 2021). By 1990, the gharial population in NCS had recovered to 1065 (all sex and size classes) in 1992 from an initial population of 107 individuals in 1979 (Hussain 1999; Singh 1985). Following the increase in gharial population, all activities under Project Crocodile, including grow‐and‐release and monitoring, were discontinued in the mid‐1990s (Nair et al. 2012) with the expectation that the population would continue to recover naturally. The recovery was short‐lived, as the gharial population dwindled again to fewer than 200 individuals by the early 2000s (Stevenson 2015). Since then, the gharial population in the Sanctuary has been continuously monitored; however, a comprehensive assessment of the population trend, nesting success and habitat use remains lacking. Therefore, in the present study, we analyzed the population trend, evaluated nesting efforts, and identified factors influencing the distribution of gharial in the Chambal River within the NCS.

## 2 Methods

### 2.1 Study Area, Permits, and Ethical Statement

**FIGURE 1:**
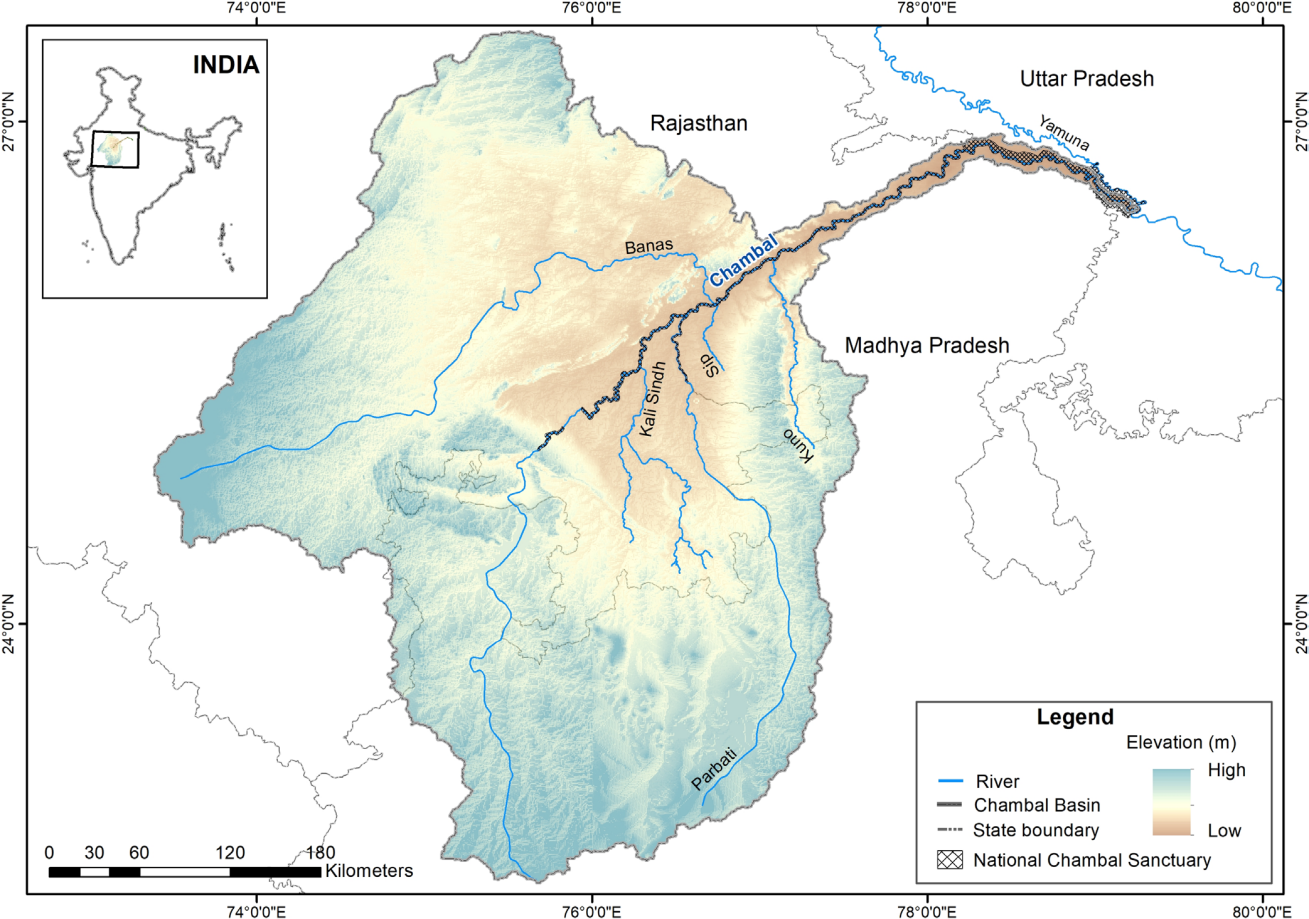
Map showing the National Chambal Sanctuary along the Chambal River, India.

The Chambal River originates in the Vindhya hill ranges in Madhya Pradesh and flows for approximately 960 km in the north‐northeastern direction and meets the Yamuna River in Uttar Pradesh (Nath 1989). Along the way, it forms the border of Rajasthan, Madhya Pradesh, and Uttar Pradesh. A 600 km stretch of the Chambal River from Jawahar Sagar dam in Rajasthan to Panchhnada at the Chambal‐Yamuna confluence in Uttar Pradesh constitutes the National Chambal Sanctuary. This riverine stretch is part of the Sanctuary and passes through Rajasthan, Madhya Pradesh, and Uttar Pradesh (Hussain and Badola 2001) (Figure 1). We surveyed ~425 km of the Chambal River between Pali (Chambal‐Parvati confluence) and Panchnada (Chambal‐Yamuna confluence) and the lower 60 km stretch of Parbati River falling within the National Chambal Sanctuary. The Forest Department of Madhya Pradesh (Letter no. 8200), Rajasthan (Letter no. 1399), and Uttar Pradesh (Letter no. 3093) provided the necessary permissions for the population survey. No animal ethical clearance was required for the study.

### 2.2 Data Collection

#### 2.2.1 Population Counts and Habitat Variables

Boat‐based visual encounter surveys were conducted during daylight hours in the winter months of February to March from 2017 to 2019. These surveys were conducted during winter months following the assumption that crocodylians, as ectotherms, are more likely to come out from the water to bask during sunny days, thereby increasing the likelihood of detecting individuals (Hussain 1999). During this period, the temperature in the study area remains optimal for basking, particularly during daylight hours (10:00–16:00 h). The survey was conducted using an aluminum boat with a 25HP outboard motor moving at a constant speed of 6–8 km/h. Observers sitting on the front of the boat were equipped with binoculars (10 × 50 magnification) to spot the basking gharial and mugger. The georeference (latitude and longitude), time, number of individuals, size class, and sex were recorded for each sighting. The size classes were recorded as hatchlings: > 0.6 m, yearlings: 0.6–0.9 m, juveniles: 0.9–1.8 m, subadults: 1.8–3.0 m and adults > 3.0 m. The habitat variables were collected at regular intervals (2017 = 2.5 km, 2018 = 1 km, and 2019 = 1 km) and whenever any gharial individual was sighted. The number of basking animals was counted using the direct count method, expressed in the number of individuals to obtain the relative density index (RDI)—individuals sighted per km of river shoreline (Bayliss 1987; Grigg and Kirshner 2015; Hussain 1999). Additionally, mugger population counts were obtained from published literature for the population forecast (Sharma et al. 2024).

#### 2.2.2 Nest Count

The gharial is a hole‐nesting species that nests exclusively in the sand (Bustard 1980; Hussain 1999), and an adult female gharial lays a nest each year (Whitaker and Basu 1982). The nesting season of gharials spans 3 months, starting in mid‐March with the laying of eggs and ending in mid‐June with the emergence of hatchlings (Hussain 1999). We counted the total number of nests each year from 2017 to 2019. Multiple surveys were conducted in synchrony with nesting events, such as egg laying, incubation, and hatching, between mid‐March and mid‐June. These surveys were carried out along the river using an inflatable boat and on foot, depending on the accessibility of nesting sites, to determine nesting count. In a series of surveys, the first survey was conducted in late March–early April to confirm the nesting sites along the riverbanks. Subsequently, regular monitoring surveys were conducted during the incubation period (April–June) to keep an account of any nest loss due to predation and disturbances. Following this, post‐hatching surveys were conducted after the hatchlings emerged in early June. Post‐hatching surveys are ideal for deriving the best estimate of the number of nests, as open cavities with broken egg shells can be easily located (Text S1).

### 2.3 Analysis of Data

#### 2.3.1 Population Trends

Monitoring population change is crucial to wildlife conservation and management (Silvy 2020). We utilized the exponential growth model to determine the population growth of the gharial population. The population growth rate parameters *r* (exponential rate of increase) and *λ* (finite rate of increase) were calculated following the equations derived by Caughley (1977). The population growth rate was determined by calculating the slope of the linear regression of counts transformed to natural logarithms, and the finite rate of increase (*λ*) was calculated by back‐transforming the exponential rate of increase (*r*) (Caughley 1977; Bayliss 1987; Hussain 1999; Silvy 2020). Additionally, we have forecasted the gharial and mugger population till 2050 based on current population trend estimates, following the exponential growth equation:

$$P_{t}=P_{o}e^{\mathrm{rt}}$$

where *P*_*t*_ is population at time *t*; *P*_*o*_ is initial population size; *r* = the growth rate, *t* = time.

#### 2.3.2 Nesting Efforts

Nest monitoring, along with other key population parameters such as abundance, distribution, size‐class composition, and population trends, is crucial for assessing the ecological status of the species (Bayliss 1987; Hussain 1999). Nest counts provide an index of abundance, which offers insights into the presence of female breeding within the population. Nest counts provide a reliable index of abundance and offer insights into the presence of breeding females within the population. Nesting success was calculated by dividing the number of predated or damaged nests by the total number of observed nests and expressing the result as a percentage. The nesting effort was determined using the formula *n/N*, where *n* represents the total number of nests observed, and *N* is the total population size. The nesting effort percentage was calculated to provide an indicative measure of reproductive output relative to the observed population during the survey period. All calculations were carried out in Microsoft Excel 2016, RStudio, version 2023.09.0 and R version 4.4.0 (R Core Team 2024).

#### 2.3.3 Winter Habitat Use

We examined the effect of habitat variables such as channel depth, channel width, and substrate types and anthropogenic stressors such as riverbank agriculture, sand mining, and fishing on gharial presence using generalized linear models (GLM). The habitat variables were selected based on a priori understanding of species ecology (Table S1). The selected variables were tested for multicollinearity using Spearman's rank correlation, and any pair of variables showing Spearman's rank correlation coefficient (Spearman's *ρ*) > 0.7 was excluded from the analysis. The continuous variables were *z‐*standardized, the data were checked for overdispersion, and an appropriate error distribution, that is, negative binomial, was used for analysis. We evaluated the habitat use using a generalized linear model (GLM) implemented in the *MuMIn* package (Barton and Barton 2015) in R version 4.4.0 (R Core Team 2024). We used corrected Akaike's information criterion (AICc ≤ 2) adjusted for the sample size to rank models and identify best‐fit models to explain the habitat use of gharial in the Sanctuary (Burnham and Anderson 2004). Furthermore, the depth preference of gharials was assessed using Manly's selection ratio (Manly et al. 2007), which quantifies habitat selection by comparing the proportional use of available habitat types. The analysis was conducted using the *adehabitatHS* package (Calenge 2006) in R version 4.4.0 (R Core Team 2024).

## 3 Results

### 3.1 Population Status and Rate of Increase

**FIGURE 2:**
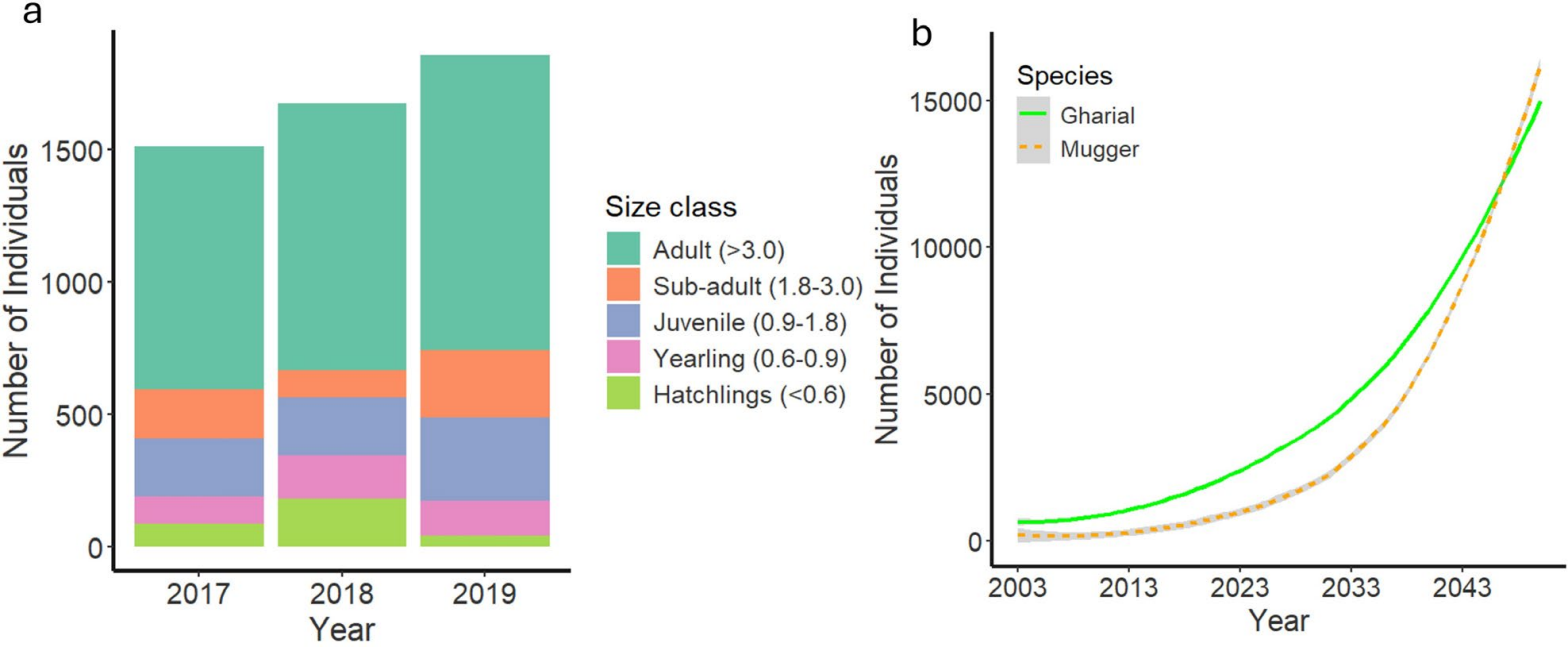
(a) Population structure of gharial ( *Gavialis gangeticus* ) in the National Chambal Sanctuary between 2017 and 2019, showing numbers across different size classes. (b) Population forecast of gharial and mugger ( *Crocodylus palustris* ) in the National Chambal Sanctuary, projecting trends over time.

The gharial population steadily increased, from 1512 individuals of all size classes in 2017 to 1857 individuals in 2019 (Table 1). The size class composition of the gharial population in the National Chambal Sanctuary from 2017 to 2019 indicates a predominance of adult individuals (> 3.0 m), contributing over 60% of the total population across all 3 years. While the overall population increased, the hatchling population (< 0.6 m) showed a significant decline, dropping from 5.8% in 2017 to 2.1% in 2019, despite a temporary spike to 10.8% in 2018. Juveniles (0.9–1.8 m) and subadults (1.8–3.0 m) exhibited an increasing trend, with juveniles rising from 14.6% to 17% and subadults from 12.2% to 13.7% over the 3 years. Yearlings (0.6–0.9 m) showed a moderate decline from 9.7% in 2018 to 7.1% in 2019 (Table 1 and Figure 2a).

**TABLE 1:** Population count and size class of gharial in the National Chambal Sanctuary during population census surveys conducted between 2017 and 2019. The percentage composition of each size class is given in parentheses.

Size class (in m)	Number of gharial individuals and size class composition		
	2017	2018	2019
Hatchings (< 0.6)	87 (5.8%)	180 (10.8%)	39 (2.1%)
Yearling (0.6–0.9)	101 (6.6%)	165 (9.7%)	133 (7.1%)
Juvenile (0.9–1.8)	221 (14.6%)	220 (13.2%)	315 (17%)
Subadult (1.8–3.0)	184 (12.2%)	100 (6%)	254 (13.7%)
Adult (> 3.0)	919 (60.8%)	1008 (60.3%)	1116 (60.1%)
Total population	1512	1673	1857

The population trend analysis of gharials between 2017 and 2019 shows an overall positive growth rate, with an overall exponential growth rate ($\bar{r}$ = 0.032) and a finite rate of population increase (*λ* = 1.032), resulting in an annual increase of 3.2%. During the study period, most size classes exhibited positive growth, with juveniles (0.9–1.8 m) and subadults (1.8–3.0 m) showing the highest increases of 11.2% and 9.4% per annum, respectively. The smallest size class (< 0.6 m) experienced a sharp decline of 37.6% per annum. The adult population (> 3.0 m) grew modestly at 2.6% per annum (Table 2).

**TABLE 2:** Relative density index (RDI—individuals sighted per km of the river) of different size classes of gharial sighted during surveys from 2017 to 2019 in the National Chambal Sanctuary, and the exponential rate of increase ($\bar{r}$), finite rate of population increase (*λ*), and per cent change per annum.

Size class	RDI			*p*	$\bar{r}$	*λ*	Change per annum (%)
	2017	2018	2019				
< 0.6	0.205	0.367	0.080	0.58	−0.472	0.624	−37.6
0.6–0.9	0.238	0.337	0.271	0.75	0.066	1.069	6.9
0.9–1.8	0.520	0.449	0.643	0.60	0.106	1.112	11.2
1.8–3.0	0.433	0.204	0.518	0.88	0.090	1.094	9.4
> 3.0	2.162	2.057	2.278	0.66	0.026	1.026	2.6
Overall	3.558	3.414	3.790	0.59	0.032	1.032	3.2

Furthermore, the comparative population forecast for sympatrically occurring gharial and mugger reveals that the mugger population will surpass the gharial population in the next two decades (Figure 2b).

### 3.2 Nesting

Nesting efforts also increased during this period, with 402 nests recorded in 2017, 421 in 2018, and 486 in 2019 (Table 3). The proportion of nests relative to the population across all size classes remained relatively stable (26.6% in 2017, 25.2% in 2018, and 26.2% in 2019), whereas the proportion of nests relative to adults (> 3.0 m) ranged between 41.8% and 43.7% during this period. However, nesting success rate showed a declining trend, with 87.5% success in 2017, decreasing to 74.1% in 2018, and further dropping to 64.6% in 2019.

**TABLE 3:** Summary of nesting success during (2017, 2018, and 2019) and proportion of nest effort within the gharial population.

Year	Population count		No. of nests		Nesting success (%)	Proportion (%)	
	All size classes	Adults (> 3.0 m)	Total	Successful		All size classes	Adults (> 3.0 m)
1979	107	—	13	—	—	12.2	—
1985	605	—	35	—	—	5.8	—
1988	820	100	52	—	—	6.3	52.0
2017	1512	919	402	352	87.5%	26.6	43.7
2018	1673	1008	421	312	74.1%	25.2	41.8
2019	1857	1116	486	314	64.6%	26.2	43.5

### 3.3 Winter Habitat Uses

We collected 1934 habitat points during the survey period 2017 (*n* = 303), 2018 (*n* = 817), and 2019 (*n* = 815), including 721 independent gharial presence points. No pair of variables tested for multicollinearity showed a significant correlation; hence, we used all variables for the analysis. We evaluated 72 models with different combinations of variables to evaluate the gharial habitat use (Table S2). The best‐supported models included substrate type (clay, rock, and sand), agriculture, and channel depth (Table 4 and Figure S1). The model indicated a negative association between gharial habitat use and clay substrate (clay1: estimate = −0.81, *p* = 0.001; clay2: estimate = −0.87, *p* = 0.001), rocky substrate (rock2: estimate = −0.60, *p* = 0.05), and channel depth (cd: estimate = −0.41, *p* = 0.001). In contrast, habitat use was positively associated with the sandy substrate (sand1: estimate = 0.95, *p* = 0.001; sand2: estimate = 1.26, *p* = 0.001). Further, Manly's selection ratio revealed gharial preference for shallow waters, with a selection ratio of 1.25 (SE = 0.15, *p* = 0.09) for depths between 0 and 1 m and 1.19 (SE = 0.08, *p* = 0.02) for depths between 1.1 and 2 m, suggesting a tendency to occupy shallower areas. However, their selection ratios decreased at greater depths, with values dropping below 1, indicating a lower preference for deeper habitats (Table S3).

**TABLE 4:** Summary of the best model obtained using the generalized linear model (GLM) to assess the habitat use of gharial in the National Chambal Sanctuary, India. Akaike information criterion of each model adjusted for small sample size (AICc), number of parameters (*k*), the difference in AICc from the best‐performing model (ΔAICc), and Akaike weight.

Variables	Estimate (SE)	*p*
Agri^1^	−0.46 (0.21)	0.05
Agri^2^	−0.58 (0.30)	ns
Channel depth	−0.41 (0.08)	0.001
Clay1^1^	−0.81 (0.19)	0.001
Clay^2^	−0.87 (0.22)	0.001
Rock^1^	−0.17 (0.22)	ns
Rock^2^	−0.59 (0.25)	0.05
Sand^1^	0.94 (0.19)	0.001
Sand^2^	1.26 (0.19)	0.001

Abbreviations: Agri^1^, agriculture on one bank; Agri^2^, agriculture on both banks; Clay^1^, clay on one bank; Clay^2^, clay on both banks; ns, not significant; Rocky^1^, rocky on one bank; Rocky^2^, rocky on both banks; Sandy^1^, sand on one bank; Sandy^2^, sand on both banks.

## 4 Discussion

Monitoring population trends is a cornerstone of wildlife conservation and management, providing essential data for informed decision‐making and evaluating the effectiveness of conservation interventions (Lindenmayer and Likens 2010). Our findings indicate an increase in the gharial population in the National Chambal Sanctuary between 2017 and 2019, likely attributed to a combination of habitat protection and population augmentation through the grow‐and‐release program. However, the observed growth rate during this period (2017–2019; $\bar{r}$ = 0.032) was lower than that reported for 1988–1992; $\bar{r}$ = 0.065 (Hussain 1999). Such a pattern of decline in growth rate is consistent with ecological principles, as no population grows indefinitely at a constant rate (Silvy 2020). Population growth in crocodylians and turtles is regulated by multiple factors, including adult density, nesting effort, resource availability, and intra‐ and interspecific competition (Brien et al. 2016; Honarvar et al. 2008; Zhao et al. 2019). Additional influences include stochastic environmental variation and anthropogenic stressors, such as incidental mortality in fishing nets. Environmental conditions, particularly temperature, rainfall and unregulated release of water during the incubation and hatching, also play a key role in embryo survival and hatching success, thereby shaping population dynamics (Somaweera et al. 2020).

Although single daylight boat‐based surveys provide valuable insights into population trends, our comparisons with earlier surveys highlight differences in gharial counts across years in the Chambal River. In comparison with earlier surveys conducted using a similar approach. Lang et al. (2018) reported 1316 gharials across size and sex classes in January 2017, and 1216 individuals in February 2017, both lower than the 1512 individuals recorded in our survey during the same period. Similarly, Lang et al. (2018) reported 1681 gharials in February 2018, which is comparable to the 1673 individuals recorded in the present study. Such differences are not unexpected, as environmental factors, including weather conditions, time of day, water levels, species behavior, and observer experience, can strongly influence detection probabilities, thereby contributing to variability in sightings (Ahizi et al. 2021; Bayliss 1987; Nair et al. 2012). These limitations highlight the need for more robust methodologies, such as mark–recapture and occupancy modeling, to obtain more accurate population estimates.

Beyond overall abundance, the demographic structure of the population provides critical insights into recruitment, survival, and long‐term viability. In the present study, the higher proportion of adult individuals (~60%) mirrors patterns observed in the wild gharial population and in other crocodylians (Choudhary et al. 2017; Hussain 1999), where smaller size classes contribute relatively little while adults dominate overall population structure (Briggs‐Gonzalez et al. 2017; Downs et al. 2015). The low representation of smaller size‐class individuals (< 0.9 m: hatchlings and yearlings) across the survey years can be attributed to multiple factors, including differential mortality, environmental variability, and detection biases (Bayliss 1987; Hussain 1999; Nair et al. 2012). Smaller size classes typically experience mortality exceeding 90% in the wild, primarily due to predation, whereas survival improves with increasing size due to reduced vulnerability and greater resilience to natural stressors (Briggs‐Gonzalez et al. 2017; Grigg and Kirshner 2015; Somaweera et al. 2020, 2019). Environmental factors such as weather conditions, time of day, water levels, the behavior of the species and observer experience can significantly influence the detection, leading to inherent variability in sightings (Ahizi et al. 2021; Bayliss 1987; Nair et al. 2012). While population counts from single daylight boat‐based surveys offer useful insights into population trends, they may not accurately represent true population size due to imperfect detection. To obtain more reliable estimates of population parameters, employing robust methodologies such as mark–recapture and occupancy models is recommended.

Habitat preferences further shape population structure. Winter habitat use suggests that gharial presence is positively influenced by sandy substrate, which provides optimal basking and nesting sites (Hussain 2009; Vashistha et al. 2021). Sandy banks are preferred due to ease of movement and thermoregulatory benefits compared to clay or rocky substrates (Hussain 2009; Katdare et al. 2011). A negative association was observed with channel depth, with gharials showing a slight preference for shallow stretches (Table S3 and Figure S1). Although deep pools are known to provide a higher cross‐sectional area and the greatest usable habitat, no clear preference for deeper classes (> 4 m) was observed, possibly due to the high fishing intensity in these pools (Das et al. 2024). Negative associations with anthropogenic stressors align with the species' known preference for undisturbed stretches of river (Katdare et al. 2011; Neupane et al. 2020; Panda et al. 2023; Yadav et al. 2022). Overall, these results suggest that gharials prefer sandy habitats while avoiding areas with clay, rocky substrates, and high agricultural intensity, highlighting the importance of maintaining sandy stretches and minimizing anthropogenic disturbances in critical gharial habitats. The habitat use described here primarily refers to winter habitat preference, as the surveys were conducted when basking is a predominant activity of gharials. Consequently, the identified habitat preferences may not fully represent year‐round patterns of habitat use, potentially introducing biases in understanding the species' overall habitat utilization. Moreover, while we recorded the presence of anthropogenic stressors such as agriculture, sand mining, and fishing, we did not quantify their intensity. Future studies should integrate such measures to more accurately assess their influence on gharial habitat use.

The growth of the Chambal gharial population has broader conservation significance, as this stronghold now serves as a source population for recolonization of rivers such as the Parbati and Yamuna (De et al. 2020; Khandal et al. 2017). Both rivers function as relatively unsuitable sink habitats, which, although easily accessible for Chambal gharials, remain less conducive for gharials due to high human stressors and lack of adequate basking and nesting habitats, such as sandy riverbanks (De et al. 2020; Khandal et al. 2017). Nonetheless, the fate of these sink populations is therefore uncertain, and extirpation is likely if the Chambal population declines. Chambal currently supports ~80% of the world's adult gharials and ~90% of wild nests (Lang et al. 2019), and it is also the principal site from which eggs are collected for the grow‐and‐release program (Sharma et al. 2021). Despite its importance, the river remains threatened by habitat degradation from riverbank agriculture, sand mining, incidental mortality in fishing nets, and reduced water flow (Hussain 2009; Katdare et al. 2011; Sharma et al. 2021). Another pressing concern is the rapidly increasing mugger population in the Sanctuary, which intensifies competition for space and resources and poses a significant threat to gharial recovery in the Sanctuary (Maharana and Mohapatra 2021; Rao and Choudhury 1992; Sharma et al. 2024). Recent documentation of juvenile gharial predation by adult muggers highlights the urgency of management interventions. Potential strategies include translocating breeding muggers or relocating nests to alternative habitats to reduce overlap in prime gharial habitats (Sharma 2024). Although our forecasts provide useful insights into potential population trajectories, they cannot be validated against more recent data. Future long‐term monitoring will therefore be essential to assess the accuracy of these projections. The population forecasts based on exponential growth models provide useful theoretical insights, but they do not incorporate key ecological constraints such as density dependence, competition with mugger, resource limitations, or stochastic environmental factors. As a result, such projections should be interpreted cautiously and viewed as heuristic tools rather than definitive predictions of long‐term trends in crocodylian populations.

Further, the reasons for the decline in the gharial population during the late 1990s remain unclear, largely due to the absence of systematic monitoring data. Nevertheless, it is evident that many of the pressures contributing to earlier declines persist, including increasing anthropogenic disturbances and the growing demand for freshwater resources (Hussain 1991; Katdare et al. 2011). These ongoing threats underscore the urgent need for conservation action through strengthened protection measures, enhanced public awareness, and active community engagement to promote the sustainable use of river resources. To ensure long‐term recovery, conservation efforts should prioritize targeted actions such as continuous monitoring of population trends and nesting activity, protection of nests and hatchlings, and, where necessary, the translocation of vulnerable nests to safer sites. These measures, combined with community‐driven conservation strategies, can significantly contribute to the recovery of gharial populations. Additionally, research on key demographic parameters such as survival and mortality rates across different size classes of both wild and reintroduced individuals is crucial to inform and improve the success of the conservation initiatives.

## Author Contributions

**Surya Prasad Sharma:** data curation (equal), formal analysis (equal), software (equal), visualization (equal), writing – original draft (equal). **Suyash Katdare:** data curation (equal), writing – original draft (equal). **Ruchi Badola:** conceptualization (equal), funding acquisition (equal), writing – review and editing (equal). **Syed Ainul Hussain:** conceptualization (equal), funding acquisition (equal), investigation (equal), writing – review and editing (equal).

## Funding

This study was funded by the Grant‐in‐Aid Fund of the Wildlife Institute of India, Dehra Dun and the National Mission for Clean Ganga, Ministry of Jal Shakti, Government of India (Grant No. B‐02/2015‐16/1259/NMCG‐WII PROPOSAL and B‐03/2015‐16/1077/NMCG‐NEW PROPOSAL). The funding agency has no role in the design of the study and the collection, analysis, and interpretation of data and in writing the manuscript.

## Conflicts of Interest

The authors declare no conflicts of interest.

## Supporting information


**Appendix S1:** ece372643‐sup‐0001‐AppendixS1.docx.

## Data Availability

The data supporting the findings of this study are available at https://figshare.com/s/8180de2a4fee636ef113.
