# Supplementary material for: Unraveling Population Trend of a Critically Endangered Freshwater Crocodylian, Gharial ( Gavialis gangeticus ) in the National Chambal Sanctuary, India
Source: Ecol Evol. 2025 Dec 10;15(12):e72643. doi: 10.1002/ece3.72643 (PMC12690217; doi:10.1002/ece3.72643)
Supplement: Supplementary file 1 — Appendix S1: ece372643‐sup‐0001‐AppendixS1.docx. [file ECE3-15-e72643-s001.docx]

*Supplemental Materials for*

**Title: Unraveling Population Trend of a Critically Endangered Freshwater Crocodylian, Gharial (*Gavialis gangeticus*) in the National Chambal Sanctuary, India**

Authors: Surya Prasad Sharma^1^, Suyash Katdare^1^, Ruchi Badola^1^ and Syed Ainul Hussain^1*^

^1^Wildlife Institute of India, P.O. Box # 18, Chandrabani, Dehra Dun, 248002, Uttarakhand, India.

***Corresponding author** - Syed Ainul Hussain, E-mail: [ainul.hussain@gmail.com](mailto:ainul.hussain@gmail.com), <https://orcid.org/0009-0009-0360-2780>

**Supplementary Text**

Text S2: Detailed methodology of nesting and hatching survey.

**Supplementary Table**

Table S1: Description of habitat variables and anthropogenic stressors used to evaluate the gharial (*Gavialis gangeticus*) habitat use in the Chambal River in India.

Table S2: List of the top 10 models. Agri=agriculture, Cl=clay, Cd= channel depth, Cw=channel width, Ro=rocky, Sa=sandy. Akaike information criterion of each model adjusted for small sample size (AICc), number of parameters (k), the difference in AICc from the best-performing model (ΔAICc), and Weight.

Table S3: Gharial depth preference at depth classes inferred using Manly’s selection ratio.

**Supplementary Figure**

**Figure S1:** Plots illustrate the predicted gharial abundance in response to various environmental and habitat-related covariates modeled using a Generalized Linear Model (GLM).

**Supplementary Text**

**Text S1:** Description of Nesting and hatching survey.

Nesting activity was monitored every year from late February onwards during 2017-2019, marking the beginning of the trial nesting period. Trial nesting is the activity when a female gharial comes up to sand banks and digs empty pits. It is understood that the female digs such 'trial nests' to gauge the suitability of the habitat by checking the quality of the substrate and other environmental factors such as temperature and humidity. This activity marks the beginning of the nesting season, and it may go on for 10-15 days or till the female is satisfied with the conditions and lays eggs. Subsequently, a rapid survey was conducted from late March-early April to identify potential nesting sites along the river. The survey was conducted using an inflatable boat and on foot, depending upon the conditions. Regular monitoring during the incubation period was carried out to keep an account of any changes regarding habitat change, disturbance, or nest loss.

Post-hatching surveys were carried out to get an accurate direct count of the total number of nests laid along the river. The direct nest count can be presented as an index of the breeding female population. Thus, the number of males counted during the population census, summed up with the number of nests, provides the number of breeding adults present in the population. The survey was conducted using an inflatable boat and on foot, depending upon the conditions. A post-hatching survey is ideal in determining the exact number of nests as hatched nests are easier to identify due to the presence of an empty cavity with broken eggshells, presence of hatchlings around the nesting site, and presence of adult gharials guarding the hatchlings.

**Supplementary Table**

**Table S1:** Description of habitat variables and anthropogenic stressors used to evaluate the gharial (*Gavialis gangeticus*) habitat use in the Chambal River in India.

| Variables | Type |
| --- | --- |
| Dependent | |
| Gharial presence | Count-Number of individuals sighted |
| Independent variable | |
| Channel depth | Continuous |
| Channel width | Continuous |
| Sand | Categorical |
| Clay | Categorical |
| Rock | Categorical |
| Agriculture* | Categorical |
| Mining* | Categorical |
| Fishing* | Categorical |

**Table S2**: List of the top 10 models. ag=agriculture, cl=clay, cd= channel depth, cw=channel width, ro=rocky, sa=sandy, mi=mining, fi=fishing, lo=longitude. Akaike information criterion of each model adjusted for small sample size (AICc), the difference in AICc from the best-performing model (ΔAICc), and weight.

| **Model** | **ag** | **cd** | **cl** | **cw** | **fi** | **lo** | **mi** | **ro** | **sa** | **df** | **logLik** | **AICc** | **delta** | **weight** |
| --- | --- | --- | --- | --- | --- | --- | --- | --- | --- | --- | --- | --- | --- | --- |
| 1 | + | -0.40 | + | NA | NA | NA | NA | + | + | 10 | -2480.66 | 4981.4 | 0 | 0.139 |
| 2 | + | -0.42 | + | NA | NA | -0.04 | NA | + | + | 11 | -2479.95 | 4982.0 | 0.61 | 0.102 |
| 3 | + | -0.41 | + | NA | NA | NA | + | + | + | 11 | -2480.39 | 4982.9 | 1.48 | 0.066 |
| 4 | + | -0.40 | + | NA | + | NA | NA | + | + | 11 | -2480.64 | 4983.4 | 1.99 | 0.051 |
| 5 | + | -0.40 | + | 0.0001 | NA | NA | NA | + | + | 11 | -2480.66 | 4983.4 | 2.00 | 0.050 |
| 6 | + | -0.43 | + | NA | NA | -0.04 | + | + | + | 12 | -2479.66 | 4983.5 | 2.06 | 0.049 |
| 7 | + | -0.43 | + | NA | NA | NA | NA | NA | + | 8 | -2483.85 | 4983.7 | 2.34 | 0.043 |
| 8 | + | -0.42 | + | NA | + | -0.045 | NA | + | + | 12 | -2479.93 | 4984.0 | 2.60 | 0.037 |
| 9 | + | -0.42 | + | -0.01 | NA | -0.046 | NA | + | + | 12 | -2479.94 | 4984.0 | 2.62 | 0.037 |
| 10 | + | -0.45 | + | NA | NA | -0.044 | NA | NA | + | 9 | -2483.11 | 4984.3 | 2.89 | 0.032 |
| 11 | + | -0.41 | + | NA | + | NA | + | + | + | 12 | -2480.37 | 4984.9 | 3.47 | 0.024 |
| 12 | + | -0.41 | + | -0.004 | NA | NA | + | + | + | 12 | -2480.38 | 4984.9 | 3.51 | 0.024 |
| 13 | + | -0.44 | + | NA | NA | NA | + | NA | + | 9 | -2483.6 | 4985.3 | 3.86 | 0.020 |
| 14 | + | -0.43 | + | NA | + | NA | NA | NA | + | 9 | -2483.68 | 4985.4 | 4.01 | 0.018 |
| 15 | + | -0.40 | + | -0.0002 | + | NA | NA | + | + | 12 | -2480.64 | 4985.4 | 4.02 | 0.018 |
| 16 | + | -0.43 | + | NA | + | -0.046 | + | + | + | 13 | -2479.64 | 4985.5 | 4.05 | 0.018 |
| 17 | + | -0.43 | + | -0.0144 | NA | -0.046 | + | + | + | 13 | -2479.64 | 4985.5 | 4.06 | 0.018 |
| 18 | + | -0.43 | + | 0.0101 | NA | NA | NA | NA | + | 9 | -2483.84 | 4985.7 | 4.34 | 0.015 |
| 19 | + | -0.46 | + | NA | NA | -0.045 | + | NA | + | 10 | -2482.84 | 4985.8 | 4.36 | 0.015 |
| 20 | + | -0.45 | + | NA | + | -0.045 | NA | NA | + | 10 | -2482.93 | 4986.0 | 4.55 | 0.014 |

**Table S3:** Gharial and mugger depth preference at depth classes inferred using Manly’s selection ratio.

| **Depth(m)** | **Gharial** | | |
| --- | --- | --- | --- |
|  | **Manly's**  **Selection ratio** | **SE** | ***p*-value** |
| 0-1.0 | 1.25 | 0.15 | 0.09 |
| 1.1-2 | 1.19 | 0.08 | 0.02 |
| 2.1-3 | 0.80 | 0.08 | 0.01 |
| 3.1-4 | 0.96 | 0.08 | 0.65 |
| 4.1.5 | 0.94 | 0.10 | 0.55 |
| 5.1-6 | 1.11 | 0.13 | 0.42 |
| 6.1-7 | 0.88 | 0.14 | 0.38 |
| 7.1-8 | 0.98 | 0.22 | 0.93 |
| 8.1-9 | 1.10 | 0.24 | 0.70 |
| 9.1-10 | 0.91 | 0.34 | 0.78 |
| >10 | 0.84 | 0.14 | 0.26 |

**Supplementary figure**
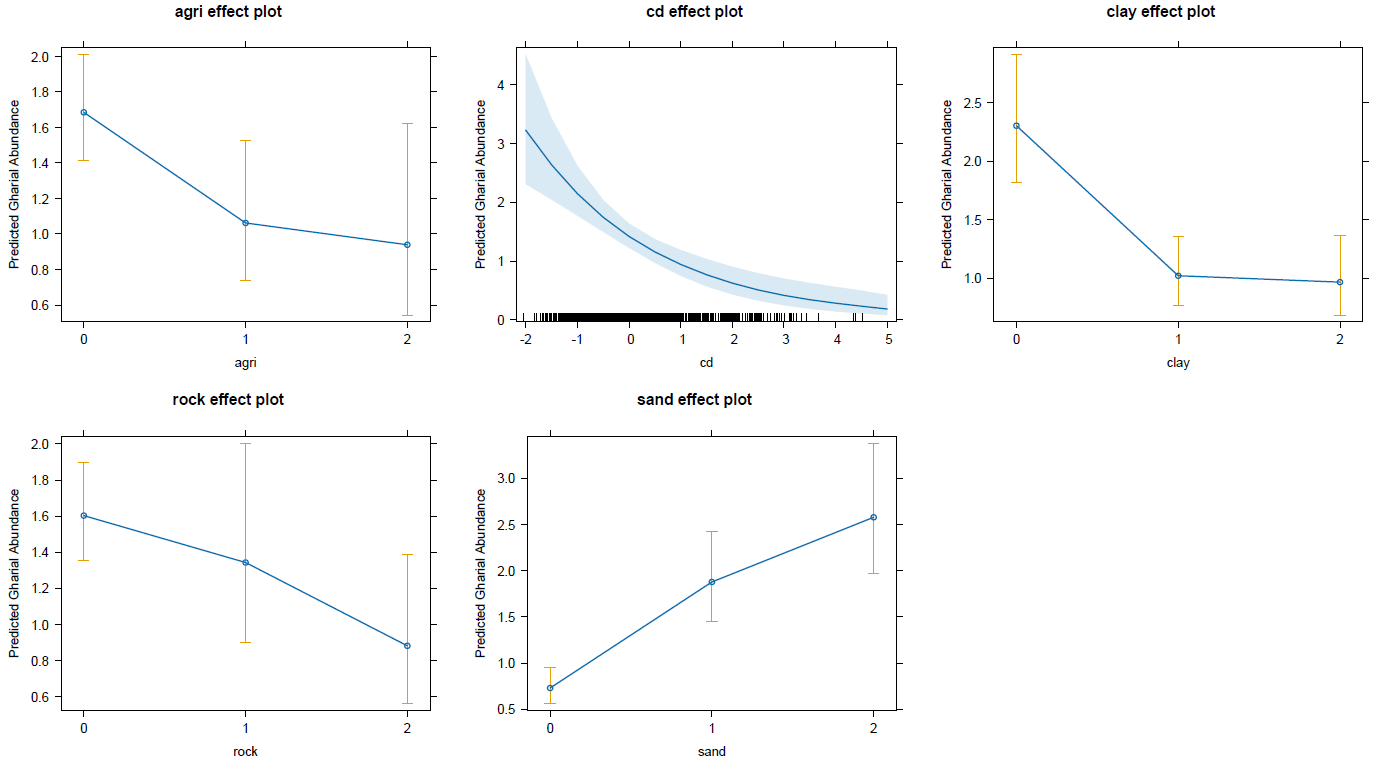


**Figure S1:** Plots illustrate the predicted gharial abundance in response to various environmental and habitat-related covariates modeled using a Generalized Linear Model (GLM).
